# Intrasubtype C superinfected individuals mount delayed and low-titer autologous neutralizing antibody responses prior to superinfection

**DOI:** 10.1186/1742-4690-9-S2-P159

**Published:** 2012-09-13

**Authors:** D Basu, C Kraft, P Campbell, M Murphy, T Yu, P Hraber, E Chomba, J Mulenga, W Kilembe, S Allen, C Derdeyn, E Hunter

**Affiliations:** 1 Emory University, Atlanta, GA, USA; 2 Los Alamos National Laboratory, Los Alamos, NM, USA; 3 Zambia Emory HIV Research Project, Lusaka, Zambia; 4 Department of Global Health, Emory University, Atlanta, GA, USA

## Background

The potential role of neutralizing antibody in protecting against intra-subtype HIV-1 superinfection remains to be understood. We compared the early neutralizing antibody responses in three individuals, who were superinfected within one year of primary infection, to ten case-matched non-superinfected controls from a Zambian cohort of subtype C transmission cases. Sequence analysis of single genome amplified full-length env showed minimal diversification in the individuals who became superinfected with the same subtype of HIV-1 within year one post-seroconversion. We hypothesized that these superinfected individuals had a muted neutralizing antibody response that elicited little pressure on the founder virus to escape.

## Methods

We molecularly cloned envs from virus at the time of seroconversion and from virus at the time point that superinfection was detected. Using a TZM-BL pseudovirus reporter assay, we tested plasma neutralization of these autologous variants over the first year of infection.

## Results

Neutralization assays showed that autologous plasma NAb titers to founder virus were low to undetectable in all three superinfected individuals prior to superinfection. In contrast, neutralizing antibodies with a median IC50 of 1:1896 were detected as early as three months post-seroconversion in non-superinfected matched controls. There was no evidence, prior to superinfection, of cross-neutralization of superinfecting variants in any of the three cases, although cross-neutralization breadth and potency to the subtype C pseudovirus reference panel was also limited in the plasma from non-superinfected individuals. Although there was a trend towards superinfected individuals having reduced levels of gp120 binding antibodies prior to superinfection compared to non-superinfected controls, this difference was not statistically significant between the groups.

## Conclusion

These data suggest that development of antibodies, as reflected in autologous neutralizing antibodies to the primary infection variants, may provide protection and decrease susceptibility to superinfection.

